# Cell type matching in single-cell RNA-sequencing data using FR-Match

**DOI:** 10.1038/s41598-022-14192-z

**Published:** 2022-06-15

**Authors:** Yun Zhang, Brian Aevermann, Rohan Gala, Richard H. Scheuermann

**Affiliations:** 1 J. Craig Venter Institute, La Jolla, CA USA; 2 Allen Institute for Brain Science, Seattle, WA USA; 3 Department of Pathology, University of California San Diego, La Jolla, CA USA; 4 Division of Vaccine Discovery, La Jolla Institute for Immunology, La Jolla, CA USA; 5 Present Address: Chan Zuckerberg Initiative, Redwood City, CA USA

**Keywords:** Cellular neuroscience, Computational biology and bioinformatics, Machine learning

## Abstract

Reference cell atlases powered by single cell and spatial transcriptomics technologies are becoming available to study healthy and diseased tissue at single cell resolution. One important use of these data resources is to compare cell types from new dataset with cell types in the reference atlases to evaluate their phenotypic similarities and differences, for example, for identifying novel cell types under disease conditions. For this purpose, rigorously-validated computational algorithms are needed to perform these cell type matching tasks that can compare datasets from different experiment platforms and sample types. Here, we present significant enhancements to FR-Match (v2.0)—a multivariate nonparametric statistical testing approach for matching cell types in query datasets to reference atlases. FR-Match v2.0 includes a normalization procedure to facilitate cross-platform cluster-level comparisons (e.g., plate-based SMART-seq and droplet-based 10X Chromium single cell and single nucleus RNA-seq and spatial transcriptomics) and extends the pipeline to also allow cell-level matching. In the use cases evaluated, FR-Match showed robust and accurate performance for identifying common and novel cell types across tissue regions, for discovering sub-optimally clustered cell types, and for cross-platform and cross-sample cell type matching.

## Introduction

Single cell transcriptomic profiling has emerged as a powerful tool to characterize the cellular heterogeneity in complex biological systems. Large collaborative consortia, including the Human Cell Atlas^1^, NIH BRIAN Initiative^2,3^, and NIH HuBMAP^4^ have adopted the unbiased single-cell/nucleus RNA-sequencing (sc/snRNA-seq) technologies to generate reference cell type atlases at single cell resolution across many organs and species at an unprecedented level of granularity. For example, a series of recent publications have reported 128 transcriptomically-distinct cell types in human primary motor cortex (M1)^5^, 116 cell types in mouse primary motor cortex (MOp)^6^, and 75 cell types in human middle temporal gyrus (MTG) neocortex^7^. The Allen Institute for Brain Science has made these comprehensive datasets available to serve as reference cell type atlases of mammalian brain regions (https://portal.brain-map.org/atlases-and-data/rnaseq).

An important role for these reference atlases is to support the matching of data from new single-cell experiments (query data) to cell types in these reference atlases using recently-developed computational methods. Azimuth is a web application for reference-based single-cell analysis following the Seurat pipeline^8^. Online iMNF is an extension of the Liger pipeline for single-cell multi-omics integration using iterative online learning^9,10^. ScArches is a deep learning strategy for mapping query datasets on top of a reference by single-cell architectural surgery^11^. The mathematical foundation of these methods are linear algebra techniques (canonical correlation analysis (CCA) for Seurat, non-negative matrix factorization (NMF) for Liger, and latent space embedding for scArches) that effectively decompose the variance–covariance structure of large data matrices to low-dimensional spaces for integrative analysis. Deep learning tools can also identify insightful patterns in these datasets, but suffer from a loss of explainability. While these methods are great tools for single-cell data integration, e.g., to produce integrated UMAP visualization for both query and reference datasets with minimal batch effects, cell type matching is a more pragmatic use case that requires not only integrating the query cells into the reference, but also being able to make a clear distinction between common and novel cell types existing in the query dataset and across the studied conditions (e.g., scRNA-seq platforms, sample types, tissue regions, etc.).

Single cell transcriptomics is a rapidly evolving field. Though a number of scRNA-seq experimental methods have been developed recently^12^, two technology platforms have become predominantly adopted—plate-based SMART-seq and droplet-based 10X Chromium. The two technology platforms are complementary to each other—SMART-seq is labor intensive but can detect more genes whereas 10X Chromium is scalable to millions of cells but tends to detect fewer genes. Combining data from both platforms would provide substantial benefits in obtaining a comprehensive understanding of transcriptomic cell types from scRNA-seq studies. In addition, the sample type used (i.e., single cell or single nucleus) is another key factor in the design of transcriptomics experiments. For example, intact whole cells can be difficult to extract in brain tissues because of the axon and dendrite structure of neuronal cells. Thus, single-nucleus RNA-seq is often used in brain cell studies as an alternative^13^. Moreover, as more data become available from multiple single cell data consortia, cross-tissue analysis will bring a more holistic characterization of cell types in biological systems. Furthermore, knowledge about cell types is also growing in spatial dimensions using single molecular fluorescence in situ hybridization (smFISH)^14,15^, MERFISH^16–18^, and other spatial transcriptomic technologies^19^, revealing the relative location of cells in tissue samples. Soon, there will be a great need for data-driven mapping of the spatial cells onto reference cell type atlases based on their transcriptional profiles. In each of these use cases, it is expected that data from different experiment platforms will differ in their distributional properties^20^, making it even more challenging to match cells assessed using different platforms. To the best of our knowledge, no cell type matching method has yet to be validated in all of these important use cases.

Previously, we reported a computational pipeline for downstream cell type analysis of scRNA-seq data combining NS-Forest^21,22^, a random forest machine learning algorithm for the identification of minimum sets of marker genes for given cell types, and FR-Match^23^, a graph-based statistical testing approach for cell type matching of query and reference datasets, and demonstrated its performance in matching cell types in simulated datasets and from overlapping brain regions^23^. We also introduced the concept of cell type “barcodes”^22,23^ using NS-Forest marker genes to visualize and characterize the distinction between different cell types^22^. NS-Forest marker gene identification also serves as a feature selection approach for FR-Match, which essentially matches the query and reference cell types based on the similarity of the cell type barcode gene expression patterns^23^.

Here, we report significant enhancements to FR-Match (v2.0), including a normalization step, a cell-to-cluster matching scheme, and an option for utilizing cosine distance, and show that the cell type barcodes provide evidence and explainability for the matching results. Importantly, we demonstrate the superior performance of FR-Match v2.0 in four real data matching use cases. Indeed, testing computational methods in many different use cases is equally important as methods development to demonstrate the extensibility and robustness of the approach. The enhanced FR-Match v2.0 was found to effectively match cell types between platforms (10X and SMART-seq; scRNA-seq and smFISH), sample types (whole cells and nuclei), and tissue regions (human M1 and MTG) and provided evidence for novel cell types and suboptimal partitioning in the clustering step. All analyses reported in this manuscript can be reproduced following the tutorials at https://jcventerinstitute.github.io/celligrate/.

## Results

### New developments of FR-Match pipeline

**Figure 1:**
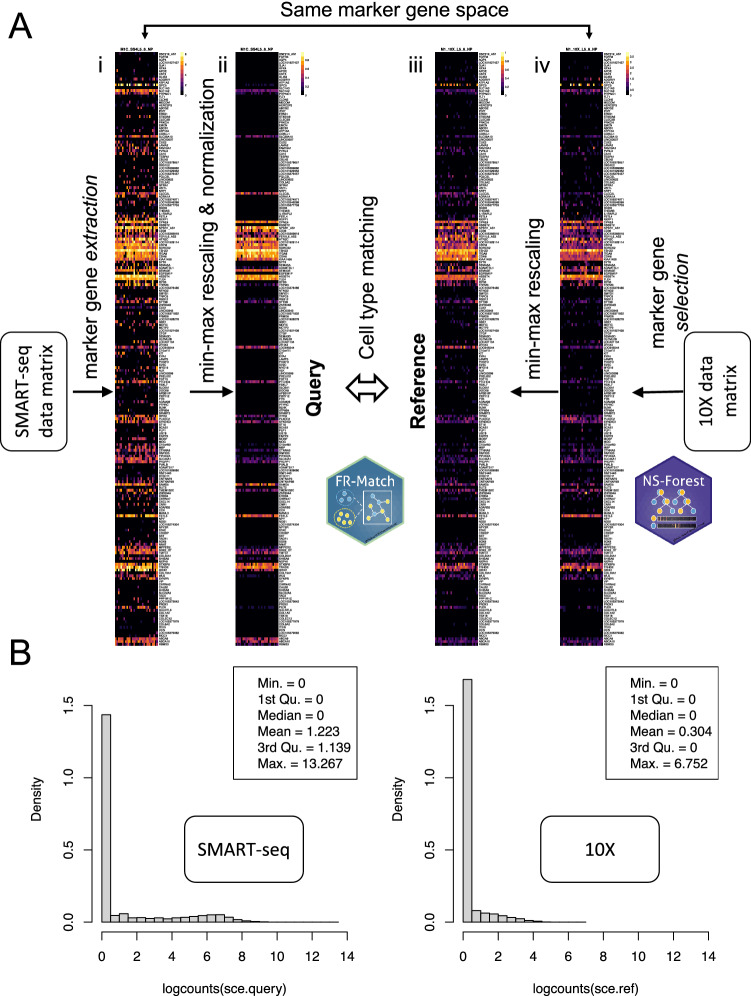
Cell type matching using FR-Match based on normalized features selected using NS-Forest. (**A**) Schematic of the cross-platform cell type matching pipeline. 10X Genomics platform scRNA-seq clustered gene expression data is used as the reference; reference marker genes are selected using the NS-Forest algorithm based on these reference data (iv); gene expression data for these reference marker genes is extracted from the query input SMART-seq data (i); platform-specific rescaling and normalization is performed; and the rescaled and normalized marker gene expression distributions of the query (ii) and reference (iii) are compared using the FR-Match algorithm. The example shown is for a matching pair of reference and query clusters. (**B**) SMART-seq and 10X data distributions. Density plots of the log_2_(CPM) data from the SMART-seq (left) and the 10X Chromium (right) platforms are shown. The SMART-seq data form a bimodal distribution, whereas the 10X data form a long-tail right-skewed distribution.

Key enhancements have been made to our previously published FR-Match pipeline^23^, motivated by important emerging cell type matching use cases. We first designed a normalization procedure based on the marker gene expression patterns observed in the cell type barcode plots, to dampen technical artifacts observed in different scRNA-seq platforms. For the two most commonly used scRNA-seq platforms—SMART-seq^24,25^ and 10X Chromium^26^, we observed that barcode plots from the SMART-seq platform (Fig. 1A(i)) and the 10X platform (Fig. 1A(iv)) showed similar marker gene expression specificity, but different expression distributions (Fig. 1B) and variable non-specific background expression. To address these technical artifacts for cell type matching, min–max rescaling is applied to each gene independently, for both SMART-seq and 10X data, to globally align the data in the range of [0, 1]. The SMART-seq platform generally shows better sensitivity for low expression genes than the 10X platform, but can also show more background noise. To reduce background noise while preserving the expression signals, the normalization step for the SMART-seq data uses a per-barcode per-gene summary statistic (mean or median) to weight the barcode pattern by multiplying the summary statistic to the gene expression submatrix of that cluster (see “Methods” section). In the case of median, the result is that for genes expressed in a minority of cells (median = 0), expression in these cells is set to zero. Finally, the weighted barcode is again rescaled to [0, 1] for matching. The above procedure effectively aligned the cross-platform barcode patterns (Fig. 1A(ii)(iii)), producing similar signal and noise levels.

To augment the original cluster-to-cluster matching in the FR-Match pipeline, an extended cell-to-cluster approach was added to FR-Match v2.0 based on an iterative procedure that allows each cell in the query cluster to be assigned a summary *p* value, quantifying the confidence of matching to a reference cluster (see “Methods” section). As a result, the cell-to-cluster and the cluster-to-cluster matching approaches differ in that the former allows different reference cell type assignments for the cells in the same query cluster, while the latter assigns the same result to all cells in the same query cluster. This extension is available as a stand-alone function FRmatch_cell2cluster() in the FRmatch R package (https://github.com/JCVenterInstitute/FRmatch).

Finally, a cosine distance option was also added for robust matching between experiments with systematic differences in data scales (see “Methods” section).

### Cell type matching between SMART-seq and 10X Chromium

**Figure 2:**
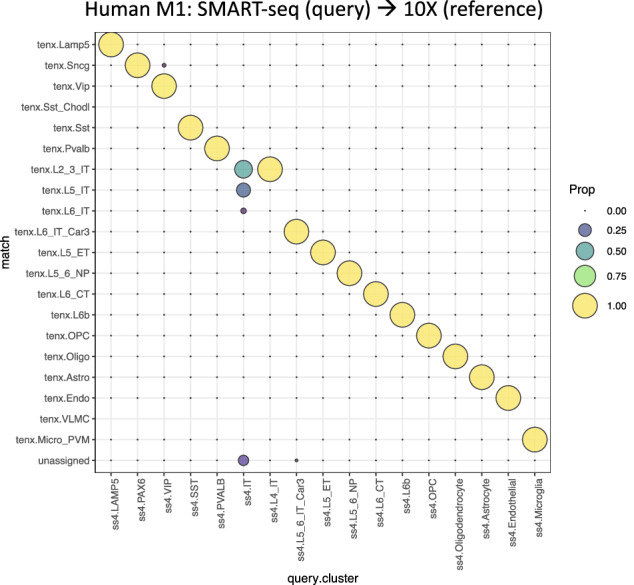
Cross-platform matching using FR-Match. Cell-to-cluster matching results for matching cell types from *SMART-seq v4* (query) to *10X Chromium v3* (reference) datasets from the human M1 brain region using FR-Match. Results are shown as the proportion of cells matched between pairs of query and reference subclass cell types. Most of the query cells are matched with the expected reference cell type subclass, aligning diagonally in the plot. The only exception is the agglomerated query IT subclass that was matched to several layer-specific reference IT subclasses or unassigned.

Using the enhanced FR-Match v2.0 pipeline and its extensions, we validated the cross-platform matching performance using Allen Institute human M1 snRNA-seq data generated using the *10X Chromium v3* protocol^5^ as the reference and an M1 snRNA-seq dataset from another Allen study on multiple human cortical regions using the *SMART-seq v4* protocol^7^ as a query. Although the raw counts of the query and the reference datasets showed very different data distributions (Fig. 1B), the distributions became more closely aligned following normalization (Supplementary Fig. 1). The FR-Match matching results produced almost all one-to-one matching at the subclass level for all query cells after normalization (Fig. 2), with the exception of the agglomerated IT query type. Due to the grouping (under-partitioning) of the layer-non-specific IT cells in the query, the majority of these cells were matched to one of two different layer-specific IT reference types. These results demonstrate that the normalization step for aligning SMART-seq and 10X data is effective and the enhanced FR-Match v2.0 is robust to perform cross-platform cell type matching between SMART-seq and 10X Chromium platforms.

To validate the fidelity of the assigned matches, we conducted a leave-one-out-cross-validation (LOOCV) analysis on these sets of data. The design of the LOOCV study is to leave one reference cluster out from the input data, so that the expected matches to the left-out reference cluster should receive “unassigned” matches, i.e., the query cell type is not represented in the reference data (a.k.a. a novel cell type). The “unassigned” match is a unique feature of the FR-Match algorithm, which determines if a query cell is not similar to any of the reference cell types based on the significance of the *p* value calculated in the algorithm (see “Methods” section). By removing each reference cluster in turn, the matched cells in Fig. 2 should be “unassigned” in the LOOCV analysis. To quantify the authenticity of the “unassigned” matches, we then calculated the accuracy and type-I error (i.e., false positivity) of the “unassigned” matches in a binary classification setting (i.e., observed positive = query cells that are expected to be matched to the left-out reference cluster, observed negative = all other query cells, and predicted positive = query cells that are matched to “unassigned”, predicted negative = query cells that are not matched to “unassigned”). The LOOCV results, together with accuracy and type-I error, are reported in Supplementary Fig. 2. In the 20 subplots in Supplementary Fig. 2A, query cells that are expected to be matched to the left-out reference cluster are dropped to the “unassigned” row at the bottom. If there are no expected matched query cells, no query cells are located in the “unassigned” row. In two subplots (Vip and L2_3_IT) where not all expected query cells are assigned to the “unassigned” row, some of the query cells are matched to a very similar reference cell type (Sncg and L5_IT, respectively), reflecting the close transcriptional similarity of these cell types. The accuracy measures are all above 99%, except for the Vip subplot with 95.41% accuracy. The type-I error levels are all below 0.05, except for the L2_3_IT and Endo subplots that have slightly elevated type-I error levels at ~ 0.08. The accuracy measure is impacted more in the large query cluster (411 cells in the query VIP cluster) where the missing true positives becoming false negatives (in this case, the number of Sncg-matched query VIP cells). Conversely, the type-I error level is impacted more in the small query clusters (32 and 11 cells in the query L4_IT and Endothelial clusters, respectively), where a single false positive will have a large impact. Overall, the LOOCV results (Supplementary Fig. 2A) and the performance measures (Supplementary Fig. 2B) show that the cross-platform matching by FR-Match is highly accurate.

### Identification of sub-optimally partitioned cell types using FR-Match

**Figure 3:**
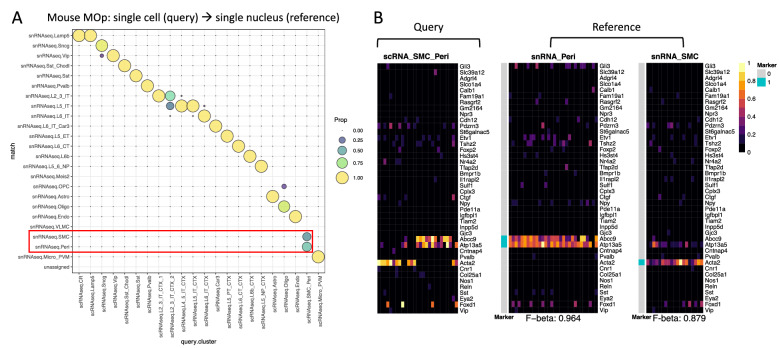
Cross-sample type matching using FR-Match. (**A**) Cell-to-cluster matching results for matching cell types from single-*cell* RNA-seq (scRNA-seq) (query) to single-*nucleus* RNA-seq (snRNA-seq) (reference) 10X datasets from the mouse MOp brain region using FR-Match. Highlighted in the red box is an example of evidence for an under-partitioned query SMC-Peri subclass in which cells were matched to either the SMC subclass or the Peri subclass in the reference dataset. (**B**) Cell type barcodes of the query SMC-Peri subclass and the corresponding reference SMC and Peri subclasses. The barcode plots clearly show two distinct expression patterns in the query cluster, each reflecting one of the two reference cluster expression patterns, supporting the under-partitioning hypothesis.

We also applied the FR-Match v2.0 pipeline to assess cross-sample type matching using a *single-nucleus* RNA-seq dataset from the Allen mouse MOp^6^ as a reference and a *single-cell* RNA-seq dataset from the MOp subset of a cell type taxonomy of the entire adult mouse isocortex and hippocampus^27^ as the query. Since both datasets were generated using the 10X protocol, we only applied the min–max scaling in the normalization step. For subclass types, most of the query types were one-to-one matched to a reference type (Fig. 3A). The highlighted box shows one exception where the query SMC-Peri cells were matched to either the SMC or Peri types in the reference, with ~ 50:50 split. In our previous simulation studies, a one-to-many match was found to indicate under-partitioning in the query cluster in some cases^23^. An examination of the cell type barcode plots for these query and reference cell types (Fig. 3B) showed two distinct patterns in the query barcode, each corresponding to one of the two reference barcodes, supporting the under-partitioning hypothesis. Thus, the FR-Match cell type matching pipeline, together with the cell type barcodes, showed excellent matching of single-nucleus and single-cell clusters, and provided solid evidence of sub-optimal partitioning based on marker gene expression in the matching results.

### Benchmark performance of other computational methods

**Figure 4:**
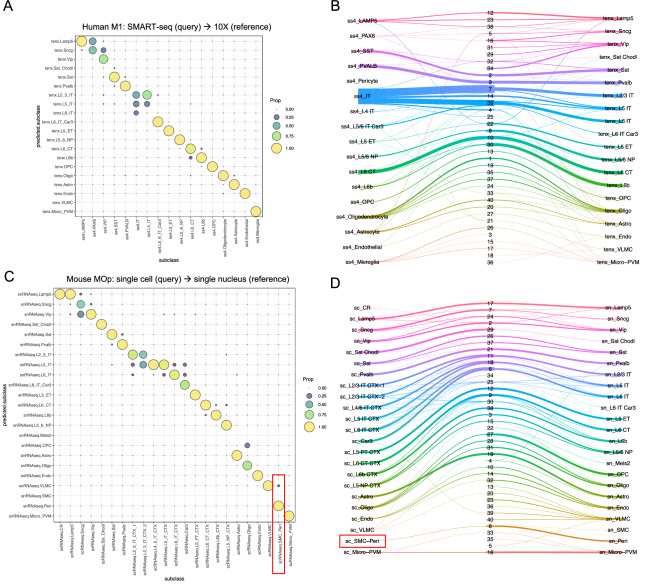
Cell type matching results using Azimuth and Online iNMF. (**A**) SMART-seq (query) to 10X (reference) matching results of human M1 subclass cell types using Azimuth. (**B**) SMART-seq (query) to 10X (reference) matching results of human M1 subclass cell types using Online iNMF. Left column: query clusters; middle column: joint clustering labels; right column: reference clusters. (**C**) ScRNA-seq (query) to snRNA-seq (reference) matching results of mouse MOp subclass cell types using Azimuth. Highlighted in the red box is the potentially under-partitioned query SMC-Peri subclass. (**D**) ScRNA-seq (query) to snRNA-seq (reference) matching results of mouse MOp subclass cell types using Online iNMF. Highlighted in the red box is the potentially under-partitioned query SMC-Peri subclass.

For the above two use cases (cross-platform and cross-sample type matching), we also benchmarked the FR-Match pipeline with the Seurat-based Azimuth^8^ and Liger-based Online iNMF^9^ computational methods using the human M1 SMART-seq to 10X (Fig. 4A,B) and mouse MOp scRNA-seq to snRNA-seq (Fig. 4C,D) matching use cases. While all cells were matched, these integration methods showed fewer clean one-to-one matchings and were not able to split the under-partitioned clusters. For example, the human M1 PAX6 query cluster was equally matched to two reference clusters—the Lamp5 and Sncg subclasses using Azimuth (Fig. 4A), while the query PAX6 cluster was exclusively matched to the Sncg reference subclass using FR-Match (Fig. 2). Using Online iNMF, no conclusive assignment of the PAX6 query cluster could be determined using its joint clustering strategy (Fig. 4B). In the mouse MOp use case, Azimuth matched all query SMC-Peri cells to the reference Peri subclass with few mismatched to the reference VLMC subclass (Fig. 4C). The Online iNMF produced joint clustering of the integrated data instead of explicitly reporting the cell-to-cell mapping. All the query SMC-Peri cells were mapped just to the Peri subclass in the reference (Fig. 4D). The deep learning method scArches has a focus on learning the latent representation of the reference atlas and outputs integrated UMAP instead of explicit matches between query and reference cells, therefore, it is not benchmarked here.

### Novel cell type detection using FR-Match

**Figure 5:**
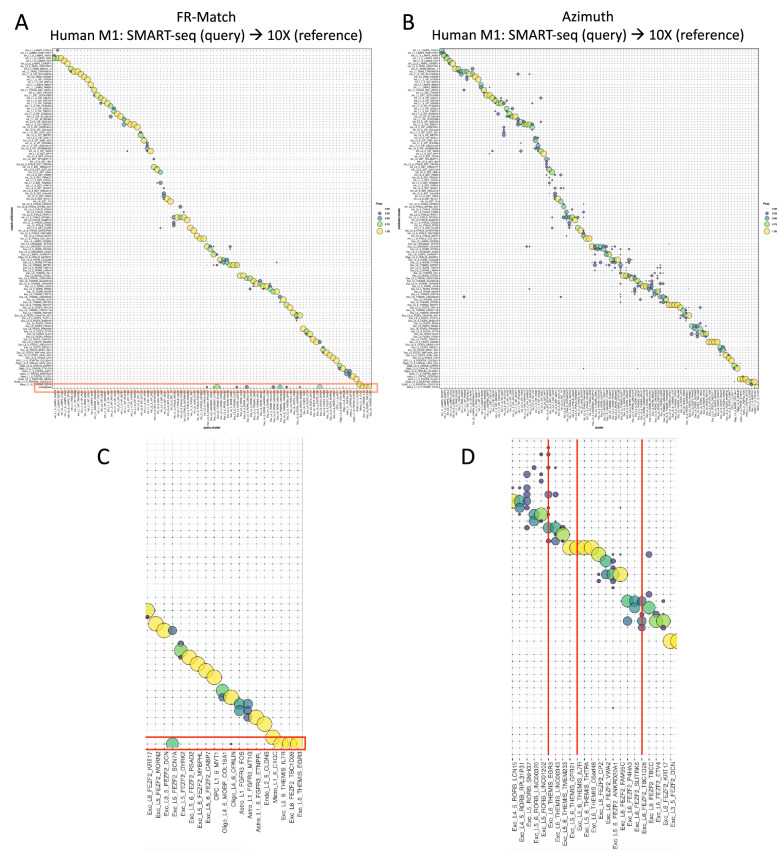
FR-Match cell type matching performance in comparison with Azimuth. (**A**) Cell-to-cluster matching results for matching cell types from SMART-seq (query) to 10X (reference) datasets of human M1 cell types at the most granular cell type resolution using FR-Match. The majority of cells in the query cell types were matched uniquely to reference cell types, showing clean diagonal matches with few off-diagonal matches. The highlighted box (in red) at the bottom is the “unassigned” row for the query cells that were not matched to any of the reference cell types based on the FR-Match results. The unassigned cells may correspond to novel query cell types not present in the reference. (**B**) Matching results for matching cell types from SMART-seq (query) to 10X (reference) datasets of human M1 cell types at the most granular cell type resolution using Azimuth. Though the majority of cells were matched along the diagonal, there were many off-diagonal matches suggesting ambiguous matching. (**C**–**D**) Enlarged view of the unassigned clusters in FR-Match results (**A**) and their corresponding columns (indicated by red vertical lines) in Azimuth results (**B**), respectively. The unassigned clusters (Exc_L5_6_THEMIS_IL7R, Exc_L6_FEZF2_TBC1D26, and Exc_L6_THEMIS_EGR3) found by FR-Match have either one-to-many matches (Exc_L6_FEZF2_TBC1D26, and Exc_L6_THEMIS_EGR3) or many-to-one match (Exc_L5_6_THEMIS_IL7R) using Azimuth.

For both use cases, we also matched the most granular cell types and benchmarked in comparison with Azimuth. In the human M1 SMART-seq to 10X use case, FR-Match produced a fairly clean diagonal matching of cell types (Fig. 5A), with several “unassigned” cell groups in the bottom row, suggesting the presence of novel cell types in the query data. Azimuth also produced a majority of matching results along the diagonal, but with many more suboptimal matches scattered off-diagonal and no indication of novel unassigned cell types in the query data (Fig. 5B). A closer look (Fig. 5C,D) shows that the unassigned clusters found by FR-Match were not uniquely matched (either one-to-many match or many-to-one match) by Azimuth, suggesting ambiguous matching results for these clusters. Similar results for the mouse MOp scRNA-seq to snRNA-seq use case can be found in Supplementary Fig. 3.

### Cell type matching between different brain regions

**Figure 6:**
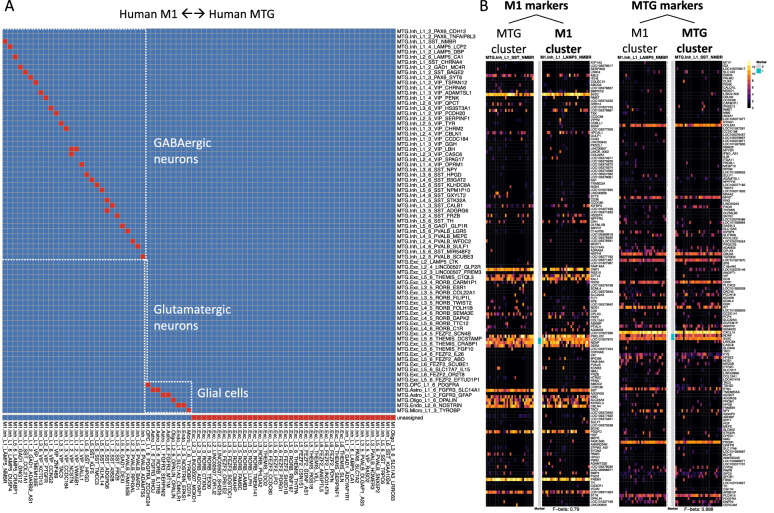
Cross-tissue region matching using FR-Match. (**A**) Cluster-to-cluster two-way matching results (red) for matching cell types across the human M1 and MTG brain regions using FR-Match. FR-Match results suggest that most of the GABAergic and glial cell types are conserved across brain regions, whereas the glutamatergic cell types appear to be region-specific. (**B**) Cell type barcodes of reciprocal marker genes. Barcode plots for matched cell types (M1.Inh_L1_LAMP5_NMBR and MTG.Inh_L1_SST_NMBR) between M1 and MTG. Matched cell types were identified by two-way FR-Match. Cyan vertical bars in between the barcodes highlight the marker genes selected for the cell types displayed. The F-beta scores of classification accuracy using the marker gene combinations are listed at the bottom. Left pair are barcodes of the two cell type clusters based on the marker genes derived from the M1 dataset. Right pair are barcodes of the two cell type clusters based on the marker genes derived from the MTG dataset. Both pairs show very similar barcode expression patterns within each pair on the reciprocal sets of marker genes, supporting the close similarity of the matched cell types between the different brain regions regardless of the marker gene sets used.

Another important matching challenge is to match cell types across different tissues or anatomic regions within a tissue. Previously, we validated the FR-Match matching performance on two anatomically-overlapping regions in human brain—cortical Layer 1 of middle temporal gyrus (MTG) and full depth (Layer 1–6) of MTG—using the bi-directional cluster-to-cluster FR-Match^23^, where all cell clusters in the Layer 1 data^28^ were found matched to cell types in the full MTG data^7^, within the specific layer expected. Here, we investigated matching results comparing two different brain regions, human M1^5^ and MTG^7^, again using the cluster-to-cluster FR-Match option. Bi-directional matching (M1 as query to MTG as reference, and vice versa) shows that most of the GABAergic inhibitory neuron types and all of the glial cell types were strongly matched across these two cortical brain regions, whereas none of the glutamatergic excitatory neuron types were matched (Fig. 6A). This suggests that the inhibitory neuron and glial cell types are conserved across brain regions, whereas the excitatory neurons are cortical region specific. Similar findings about the regional specificity of brain cell types were also reported in a scATAC-seq study of chromatin landscape in adult mouse cerebrum^29^.

We also examined the cell type barcode plots for pairs of matched cell types (e.g., Fig. 6B). The barcodes showed highly similar expression patterns of the matched types using reciprocal marker genes, even though the best marker gene sets selected for each brain regions may be different since they are defined based on the cell types present in the dataset used for marker gene selection. This also validates the robustness of the informative marker genes found by NS-Forest across experiments.

### FR-Match for spatial transcriptomics cell type calling

**Figure 7:**
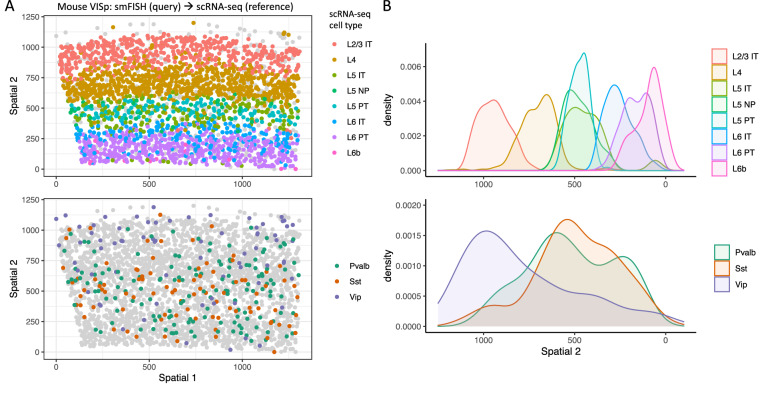
Cell type calling of spatial transcriptomics data using FR-Match. (**A**) Spatial distribution (y-axis is distance from pia) of cell types assigned for the mouse VISp smFISH dataset using scRNA-seq-defined reference cell types of the same brain region and the FR-Match cell-to-cluster algorithm. The assigned excitatory cell types clearly recapitulate the laminar distributions in the spatial coordinates (top); the assigned inhibitory cell types show the expected scattered spatial distributions. (**B**) Spatial distributions (x-axis is distance from pia) of the excitatory cell types (top) and inhibitory cell types (bottom) summarized from the FR-Match cell type assignment results for the smFISH data shown in (**A**).

Finally, FR-Match v2.0 was used for matching spatial transcriptomics cells generated by single molecular fluorescence in situ hybridization (smFISH)^30^ data to a SMART-seq scRNA-seq dataset as the reference, both from mouse primary visual cortex (VISp)^31^. De novo clustering of the smFISH data using the Scanpy pipeline^32^ and Leiden clustering algorithm^33^ with resolution 0.8 was used to produce 16 broadly-defined smFISH cell type clusters. Eleven inhibitory and excitatory neuron types transcriptomically-defined at the subclass level were considered most appropriate as the reference for matching given the level of resolution of the spatial data. Probe genes in the panel design of the smFISH protocol were used as the matching feature space, instead of using NS-Forest marker genes. The FR-Match v2.0 cell-to-cluster pipeline was used to assign a reference cell type to each spatial cell. The FR-Match results successfully recapitulated the clear laminar distributions of excitatory neurons, corresponding to the laminar distribution of their assigned cell types (Fig. 7). In contrast, the inhibitory neurons were scattered across all layers, with the Vip type located more densely in upper layers and the Sst and the Pvalb types located more densely in deeper layers as observed in previous studies^34^. Thus, the FR-Match cell type assignment for the spatially resolved cells reflected their expected laminar patterns.

## Discussion

In this manuscript, we report our extended FR-Match v2.0 pipeline to perform both cell-to-cluster and cluster-to-cluster matching with compatible normalization procedures for cell type matching across various conditions. The added normalization step and cosine distance option allow FR-Match to perform robust and accurate cell type matching across platforms (SMART-seq with 10X), sample types (single-cell with single-nucleus), brain regions (M1 with MTG), and spatial modalities (spatial transcriptomics with scRNA-seq). Compared with other methods, FR-Match effectively detected sub-optimally partitioned clusters from the previous clustering step, and uniquely identified potentially novel cell types in the query data as “unassigned” to the reference. Assessment of the “unassigned” designation was performed via leave-one-out-cross-validation, showing a median accuracy above 99%. A similar cross-validation assessment performed using Seurat, which is the core method of Azimuth, showed a median accuracy below 90%^23^. The default dimensionality reduction step provided by the NS-Forest algorithm increases the explainability of the FR-Match computational pipeline, producing cell type barcode plots that are useful for interpreting the underlying transcriptomic drivers of the FR-Match results, and thereby suggesting future research directions.

Though the core statistical method used in FR-Match is unchanged in v2.0, the enhancements described in this manuscript are critical for the method to be effective in these real-life matching use cases. The original method was only validated using one scRNA-seq platform (SMART-seq) and dataset, since scRNA-seq data had not been generated at scale at that time. As other aspects of the single cell genomics research field evolve, the computational methods also need to evolve to address the new challenges that emerge. The work described in this manuscript was ultimately motivated by these evolving downstream use cases. Indeed, there is now a great need for flexible computational tools that are able to characterize and identify cell types across assay platforms, sample types, and tissue regions. Without the enhancements included in FR-Match v2.0, it would be very difficult for users to apply FR-Match to these new problems. In this manuscript we also show a promising perspective of FR-Match in the interpretation of spatial transcriptomics data and it’s integration with atlases constructed from scRNA-seq experiments^19^.

As the single cell community expands and the reference cell type atlases becoming mature, the computational focus of scRNA-seq data analysis will also need to pivot to applications that leverage the usage of these data resources. FR-Match will play an essential role to facilitate the incremental growth of reference cell types in the emerging single cell data- and knowledgebase community resources. While four important use cases using data from healthy tissues were described in this manuscript, we would expect FR-Match to also be useful for comparative analysis between healthy and disease conditions, e.g., to detect disease-specific cell types as novel unassigned clusters or identifying transitional cell phenotypes evolving during disease progression as non-optimal matching. In addition to this comparative analysis, the described computational workflow is also being used in creating data-driven ontology and semantic representation of cell types for knowledge curation and query^35^. The explainability of cell type classification derived from the necessary and sufficient marker genes selected by NS-Forest and the cell type barcode visualization have also been engineered into the new Cell Type Card infrastructure (https://knowledge.brain-map.org/celltypes) for mammalian brain cell type classification^36^.

The size of typical single cell datasets are quickly approaching millions of cells, which results in a common challenge of computational scalability for all computational methods. Some methods, for example, the online iMNF approach, specifically addresses the integration of millions of cells by streaming a single copy of a large dataset over the internet using online learning^9,10^. Though it was not the focus of this study, the computational complexity of FR-Match is on the order of the number of query and reference cell type clusters to be compared. All analyses reported in this study were conducted in an 8-core MacBook Pro laptop with 2.8 GHz Quad-Core Intel Core i7 processor. As a guideline, it took 7 min for FR-Match to finish the job of matching the human M1 datasets with 5955 query cells and 24,526 reference cells, and 12 min for matching the mouse MOp datasets with 5666 query cells and 36,193 reference cells conducted in this study. Cell type matching of datasets with millions of cells and thousands of cell type clusters with FR-Match is currently being evaluated.

## Methods

### FR-Match cell-to-cluster matching algorithm

As originally conceived, FR-Match v1.0 is a cluster-to-cluster matching algorithm that utilizes a graphical model and minimum spanning trees to determine the data distributional equivalence between two cell type clusters derived from single-cell or single-nucleus RNA-sequencing (scRNA-seq) data in multivariate space^23^. The required input data for FR-Match are cell-by-gene expression matrices and cell cluster membership labels for both query and reference data. The output of the original FR-Match v1.0 are matching results between query and reference clusters, thus assigning known reference cell types to the query cell clusters, or defining a query cluster as an “unassigned” novel cell type not found in the reference.

Here, we extend the FR-Match algorithm (v2.0) to map each query *cell* to the known cell type clusters in the reference, i.e., cell-to-cluster matching. The input data are the same as before. If candidate query clusters are unavailable, cell type clusters can be produced using the popular Louvain^37^ or Leiden^33^ clustering algorithms for scRNA-seq data prior to matching using FR-Match.

The extended cell-to-cluster FR-Match algorithm is implemented in the function FRmatch_cell2cluster(), and its plotting function implemented as plot_FRmatch_cell2cluster() in the FRmatch R package. The steps of the algorithm and the corresponding arguments in the functions are as follows:

1. Dimensionality reduction:
  1.1. Select informative marker genes using the companion marker gene selection algorithm—NS-Forest—or user-defined marker genes for the reference dataset;
  1.2. Extract the expression data for the reference marker genes in the query dataset, i.e., project the query data into the reference feature space for reduced dimensionality;
2. Pairwise iterative matching:
  2.1. For each pair of query ($j$) and reference ($k$) clusters:
    2.1.1. For subsample iteration index $i$ iterating from 1 to the total number of iterations (subsamp.iter = 2000):
      2.1.1.1. Subsample the same number of cells (subsamp.size = 10) from the query and reference clusters, denoted as ${S}_{i}$ for the set of selected query cells;
      2.1.1.2. Perform Friedman–Rafsky test (FR test)^38^, a nonparametric statistical test for multivariate two-group comparison, and obtain *p* value from the test, denoted as ${p}_{i}$;
      2.1.1.3. Assign the *p* value to the selected query cells, i.e., ${p}_{ck}={p}_{i}$ for $c\in {S}_{i}$ and reference cluster $k$;
      2.1.1.4. Repeat 2.1.1.1 and 2.1.1.2, and obtain $p_{i^{\prime}}$ for the updated iteration $i^{\prime}$;
      2.1.1.5. Update $p_{ck} = {\text{max}}\{ p_{ck} , p_{i^{\prime}} \}$ for $c \in S_{i^{\prime}}$ and reference cluster $k$, i.e., re-assign ${p}_{ck}$ if $p_{i^{\prime}}$ is greater than previously assigned ${p}_{ck}$;
    2.1.2. End looping over iterations;
  2.2. End looping over query-and-reference-cluster-pairs;
  2.3. Obtain a *p* value matrix $\{{p}_{ck}\}$ for every query cell $c$ and reference cluster $k$;
  2.4. Apply multiple hypothesis testing correction to the *p* values (p.adj.method = “BH”);
  2.5. Determine the matched cell type for a query cell as the reference cell type that gives the maximum *p* value for that query cell, or “unassigned” (i.e., no matched cell type) if the maximum *p* value is below the *p* value threshold (sig.level = 0.1).

Though the cell-to-cluster approach is an iterative procedure, in this implementation, we utilized the pbmcapply R package^39^ to allow parallel computing using multiple cores in either local machine or grid computer settings. By default, without specifying the number of cores to use (numCores = NULL), the algorithm automatically detects the maximum number of cores in the machine and uses all cores to run the algorithm. For example, it took 7 min using default parameters to finish the job of matching the human M1 datasets with 5955 query cells and 24,526 reference cells, and 12 min for matching mouse MOp datasets with 5666 query cells and 36,193 reference cells, on an 8-core MacBook Pro laptop with 2.8 GHz Quad-Core Intel Core i7 processor.

### Difference between cell-to-cluster and cluster-to-cluster matching

The cell-to-cluster matching option provides a more flexible computational scheme for matching at single cell resolution in comparison with the more conservative two-way cluster-to-cluster matching (e.g., Fig. 6). Though the core statistical method based on the Friedman-Rafsky non-parametric multivariate test used by these two matching approaches is the same, the computational schemes of how the test is adapted to the scRNA-seq problem to perform cell-level (cell-to-cluster) and cluster-level (cluster-to-cluster) cell type matching are different. In the cell-to-cluster matching scheme, the result output is a $(C\times K)$-dimensional matrix of matching *p* values, where $C$ is the number of query cells and $K$ is the number of reference clusters; in the cluster-to-cluster matching scheme, the result output is an $\left( {L \times K} \right)^{\prime}$-dimensional matrix of matching *p* values, where $L$ and $K$ are the number of query and reference clusters, respectively. In brief, the elements in the cell-to-cluster result matrix are dynamically updated in the subsampling iteration procedure (Step 2 above), which means that when a more similar subset of cells are selected and matched to a reference cell type with a higher *p* value, the assignment of cell type for the selected cells are updated and the higher *p* value is recorded. The elements in the cluster-to-cluster result matrix are the median average from the iterations, which is equivalent to assigning the same median *p* value to all cells in the same query cluster. A detailed computational scheme design of the cluster-to-cluster approach can be found in^23^. In summary, the cell-to-cluster approach is a flexible scheme that evaluates the matching of each individual cell the query clusters as a guide, while the cluster-to-cluster approach is appropriate when the query clusters are to be considered as a whole.

### Visualization of the cell-to-cluster results

As mentioned above, the cell-to-cluster option results in a $(C\times K)$-dimensional matrix, where $C$ can be hundreds of thousands of cells, which may be less useful for the end users. In the R package, we provide a visualization function plot_FRmatch_cell2cluster(), which directly takes in the output from the FRmatch_cell2cluster() function and summarizes the results in a visually clean plot. In the cell-to-cluster plot (e.g., Fig. 2), columns are the query clusters and rows are the reference clusters, which is consistent with the orientation of the cluster-to-cluster plot (e.g., Fig. 6). The circles (both filling color and circle size) reflect the proportion of cells in the query cluster that are matched to the reference cluster; thus, the sum of the proportions for each column (a.k.a. query cluster) equals to 1. A legend to calibrate the circles is provided to the right of the plot in the default setting. With the return.value = TRUE option, the plot function also returns the matrix of proportions being plotted in the cell-to-cluster plot.

Here, we briefly describe how the proportions are calculated; more details can be found in the help page of the functions. From the $(C\times K)$-dimensional matrix of *p* values, row-wise maximum *p* values are extracted as the matching confidence score metric for each query cell and the corresponding column (a.k.a. reference cluster) names are recorded as the final matches. Query cells are grouped by query clusters; and the proportions of cells per query cluster matched to the reference clusters are calculated based on the grouped results. To be noted, columns in the $(C\times K)$-dimensional matrix are the reference clusters, and columns in the corresponding visualization (i.e., the cell-to-cluster plot) are the query clusters, which may cause confusion, but allows straightforward comparison between the cell-to-cluster and the cluster-to-cluster plots.

### Normalization

The plate-based SMART-seq and droplet-based 10X Genomics Chromium protocols are known to have very different read count distributions and detection limits^12^. Thus, normalization is a key step for performing matching across these platforms. In our pipeline, we designed a rescaling and normalization procedure based on the expression value distributions and the signal-to-background-noise patterns observed in cell type barcode plots.

First, we observed that the gene expression values of the SMART-seq and 10X data had very different dynamic ranges (Fig. 1B). The marker genes displayed in the cell type barcode were selected by the NS-Forest marker gene selection algorithm that preferentially selects binary expression genes^22^, i.e., those genes that are highly expressed in the target cell type and have little to no expression in other cell types. For the purpose of cross-platform comparison, we designed a gene-wise min–max rescaling step to align the dynamic range of gene expression of both protocols in the range of [0, 1]. Let ${{\varvec{x}}}_{g}$ be a length-$N$ vector of the expression value of marker gene $g$ across all $N$ cells in the dataset. The rescaled expression vector is:

$$ \tilde{\user2{x}}_{g} = \frac{{{\varvec{x}}_{g} }}{{{\text{max}}\left( {{\varvec{x}}_{g} } \right)}}. $$

Second, due to the higher sensitivity of the SMART-seq protocol and low detection rate of the 10X protocol for weakly expressed genes, the cell type barcodes displayed some weak signals for the genes that are not the marker genes of the given cell type in the SMART-seq data, whereas the cell type barcode of the 10X data more often displayed zero expression for those genes. For the purpose of cell type matching, the weak expression in the SMART-seq cell type barcodes can be considered a kind of background noise in its expression pattern (Fig. 1A). In order to eliminate such background noise in the SMART-seq barcode, we designed the following normalization step. Let  ${\tilde{X }}_{b}$ be the rescaled but unnormalized expression sub-matrix displayed in a cell type barcode $b$. ${\tilde{X }}_{b}$ is an $m\times {n}_{b}$ matrix, where $m$ is the number of all marker genes, and ${n}_{b}$ is the number of cells of cell type $b$. The normalized values are:

$${X}_{b}^{normalized}={{\varvec{w}}}_{b}\cdot {\tilde{X }}_{b}$$

where ${{\varvec{w}}}_{{\varvec{b}}}$ is a weighting vector consisting of the row means (or medians) of ${\tilde{X }}_{b}$. Due to the binaryness of NS-Forest marker genes, ${{\varvec{w}}}_{b}$ is usually a binary vector with values either close-to-0 or close-to-1. Due to the weighting, the dynamic range of the normalized values may shrink from [0, 1]. A final rescaling step is added to realign the maximum value of the dynamic range back to 1 sub-matrix-wise, which is:

$${X}_{b}^{final}=\frac{1}{\mathrm{max}\left({X}_{b}^{normalized}\right)}\cdot {X}_{b}^{normalized}.$$

The final expression matrix for the input of the algorithm is the column-concatenation of ${X}_{b}^{final}$ for all $b$’s, where $N={\sum }_{b}{n}_{b}$.

The above procedure is implemented in the normalization function normalization() in the FR-Match R package. In the matching use cases presented, the weighting normalization procedure was only applied in the case of cross-platform matching between SMART-seq and 10X protocols. If both the query and reference data are generated using the same platform, the weighting step is not necessary, which can be turned on or off by specifying norm.by = “mean”, norm.by = “median”, or norm.by = NULL options in the normalization() function. The effects of normalization on the data distributions using the different norm.by= options are shown in Supplementary Fig. 1.

### Cosine distance metric in FR-Match

To make matching more robust to systematic scaling difference in expression distributions, we modified the FR-Match algorithm to calculate the cosine distance that is invariant to scaling as an option for constructing the minimum spanning tree used in the FR test instead of Euclidean distance. Let ${\varvec{x}}={\left({x}_{g}\right)}_{g=1}^{p}$ and ${\varvec{y}}={\left({y}_{g}\right)}_{g=1}^{p}$ be two cells in the $p$-dimensional feature space of marker genes $g=1,\dots ,p$. The cosine similarity between the two cells is defined as:

$$\mathrm{similarity}=\mathrm{cos}\left(\theta \right)=\frac{\sum_{g=1}^{p}{x}_{g}\cdot {y}_{g}}{\sqrt{\sum_{g=1}^{p}{x}_{g}^{2}}\cdot \sqrt{\sum_{g=1}^{p}{y}_{g}^{2}}}$$

where $\theta$ is the angle between vectors ${\varvec{x}}$ and ${\varvec{y}}$. Intuitively, if the angle $\theta$ is small, then $\mathrm{cos}(\theta )$ is large, which means the two cells ${\varvec{x}}$ and ${\varvec{y}}$ are more similar to each other as the angle between their representing vectors becomes smaller in the multi-dimensional space. If two cells are from different platforms, say ${\varvec{x}}$ is SMART-seq data and ${\varvec{y}}$ is 10X data, the scale difference between their expression range is normalized by the denominator in the above equation, which is the product of the lengths of the two vectors. Finally, the cosine distance is defined as:

$$\mathrm{distance}=1-\mathrm{cos}\left(\theta \right).$$

It is suggested to use the scaling-invariant cosine distance for more robust cell type matching across platforms. The option of using cosine distance can be turned on or off by specifying use.cosine = TRUE in the FRmatch() or FRmatch_cell2cluster() functions.

To illustrate the effectiveness of using cosine distance, we conducted simulation studies in which the location and/or shape of the clusters were altered in the underlying multivariate data distribution before matching. Without loss of generality, consider multivariate random variables ${\varvec{X}},{\varvec{Y}}\sim MV{N}_{40}\left({\varvec{\mu}},{\varvec{\Sigma}}\right)$, where $MVN\left(.,.\right)$ is a Multivariate Normal distribution, ${\varvec{\mu}}\in {\mathbb{R}}^{40}$ is the location parameter and ${\varvec{\Sigma}}\in {\mathbb{R}}^{40\times 40}$ is the covariance matrix controlling the shape of the distribution. That is to say, the simulated data were generated from a $p$-dimensional Multivariate Normal (MVN) distribution with $p=40$. Here, $p=40$ is chosen because it is doubling the dimensionality evaluated in the original FR test paper^38^, accounting for the higher dimensionality of data nowadays; $p>40$ is also allowed but will serve the same purpose. The null hypothesis is ${H}_{0}:{F}_{{\varvec{X}}}={F}_{{\varvec{Y}}}$, where ${F}_{{\varvec{X}}}=MV{N}_{40}\left({{\varvec{\mu}}}_{{\varvec{X}}},{{\varvec{\Sigma}}}_{\mathbf{X}}\right)$ and ${F}_{{\varvec{Y}}}=MV{N}_{40}\left({{\varvec{\mu}}}_{{\varvec{Y}}},{{\varvec{\Sigma}}}_{\mathbf{Y}}\right)$ are the distributions of multivariate random variables ${\varvec{X}}$ and ${\varvec{Y}}$, respectively; the alternative hypothesis is ${H}_{1}:{F}_{{\varvec{X}}}\ne {F}_{{\varvec{Y}}}$. We designed three scenarios where there is location difference (${{\varvec{\mu}}}_{{\varvec{X}}}\ne {{\varvec{\mu}}}_{{\varvec{Y}}}$), shape difference (${{\varvec{\Sigma}}}_{{\varvec{X}}}\ne {{\varvec{\Sigma}}}_{{\varvec{Y}}}$), and both location and shape differences. Let $m$ and $n$ be the sample sizes of data drawn from ${F}_{{\varvec{X}}}$ and ${F}_{{\varvec{Y}}}$, respectively. We evaluated small ($m=n=20$) and large ($m = n = 100$) sample sizes. Supplementary Figure 5 shows the simulation performance (ROC curve and AUC statistic) of the FR test using either the default Euclidean distance or the cosine distance option. In all scenarios, the FR test using the cosine distance produced better ROC curves and higher AUC values compared to the standard FR test, suggesting more robust performance using the scaling-invariant cosine distance.

## Supplementary Information

Supplementary Information.

## Data Availability

All datasets used in these studies are publicly available in the Allen Brain Map Cell Types Database: RNA-Seq Data (https://portal.brain-map.org/) and NeMO Data Archive (https://nemoarchive.org/). Each dataset can be downloaded from the following list. Human M1 10X: https://portal.brain-map.org/atlases-and-data/rnaseq/human-m1-10x; Human M1 SMART-seq: https://portal.brain-map.org/atlases-and-data/rnaseq/human-multiple-cortical-areas-smart-seq; Mouse MOp single-nucleus RNA-seq: https://assets.nemoarchive.org/dat-ch1nqb7; Mouse MOp single-cell RNA-seq: https://portal.brain-map.org/atlases-and-data/rnaseq/mouse-whole-cortex-and-hippocampus-10x; Human MTG SMART-seq: https://portal.brain-map.org/atlases-and-data/rnaseq/human-mtg-smart-seq; Mouse VISp single-cell RNA-seq and smFISH: https://portal.brain-map.org/atlases-and-data/rnaseq/data-files-2018. Raw count matrices were downloaded and preprocessed by log-transformation of the count per million (CPM) data. Log(CPM) data were the input data for the FR-Match algorithm.

## Code availability

Open-source software packages—NS-Forest and FR-Match—are available in GitHub repositories. Reproducible analysis notebooks are also available as tutorials in the software GitHub page. All details can be found in https://jcventerinstitute.github.io/celligrate/.
