# Supplementary material for: Cell type matching in single-cell RNA-sequencing data using FR-Match
Source: Sci Rep. 2022 Jun 15;12:9996. doi: 10.1038/s41598-022-14192-z (PMC9200772; doi:10.1038/s41598-022-14192-z)
Supplement: Supplementary file 1 — Supplementary Information. [file 41598_2022_14192_MOESM1_ESM.pdf]

Supplementary Figures
for
Cell type matching in single-cell RNA-sequencing data using FR-Match

Authors: Yun Zhang[1], Brian Aevermann[1], Rohan Gala[2], Richard H. Scheuermann[1,3,4,*]

Affiliations: [1]J. Craig Venter Institute, La Jolla, CA, USA; [2]Allen Institute for Brain Science, Seattle, WA, USA; [3]Department of Pathology, University of California San Diego, La Jolla, CA, USA; [4]Division of Vaccine Discovery, La Jolla Institute for Immunology, La Jolla, CA, USA

***Corresponding author:** Richard H. Scheuermann

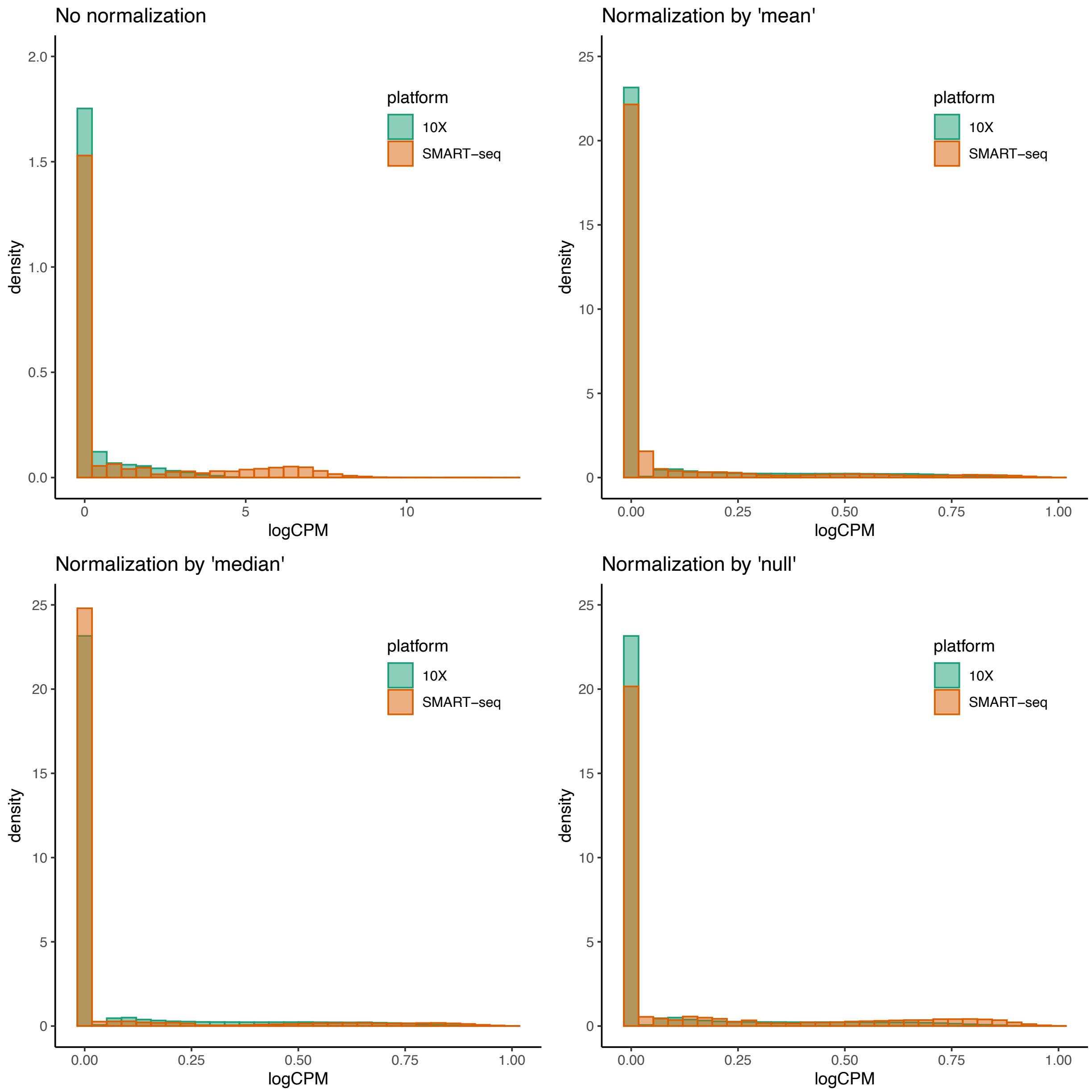

Supplementary Figure 1: Effects of the normalization options on pre- and post-normalization data distributions. The ‘No normalization’ panel is the overlay plot of distributions shown in Figure 1B. The other three panels show the post-normalization distributions for available `norm.by=` options.

A

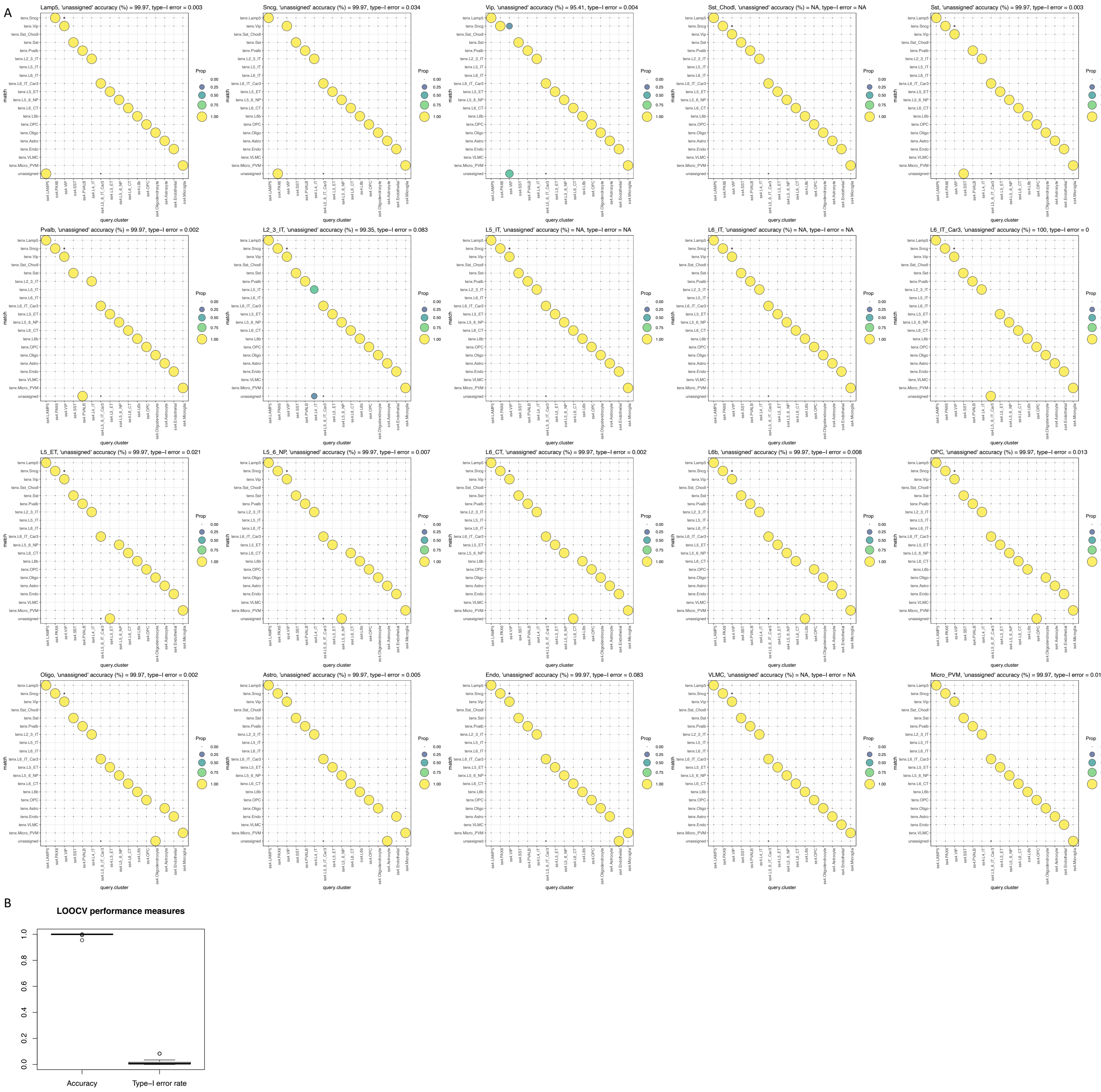

Supplementary Figure 2: Leave-one-out-cross-validation (LOOCV) results. A. LOOCV on the human M1 SMART-seq and 10X cross-platform matching using FR-Match v2.0. In each subplot, the left-out reference cluster is listed in the title, followed by performance measures of accuracy and type-I error level. When no query cluster is expected to be matched to the left-out reference cluster, the performance measures are not available (i.e., NA). **B.** Boxplot of the performance measures from panel A. Median accuracy = 99.97%, median type-I error level = 0.006.

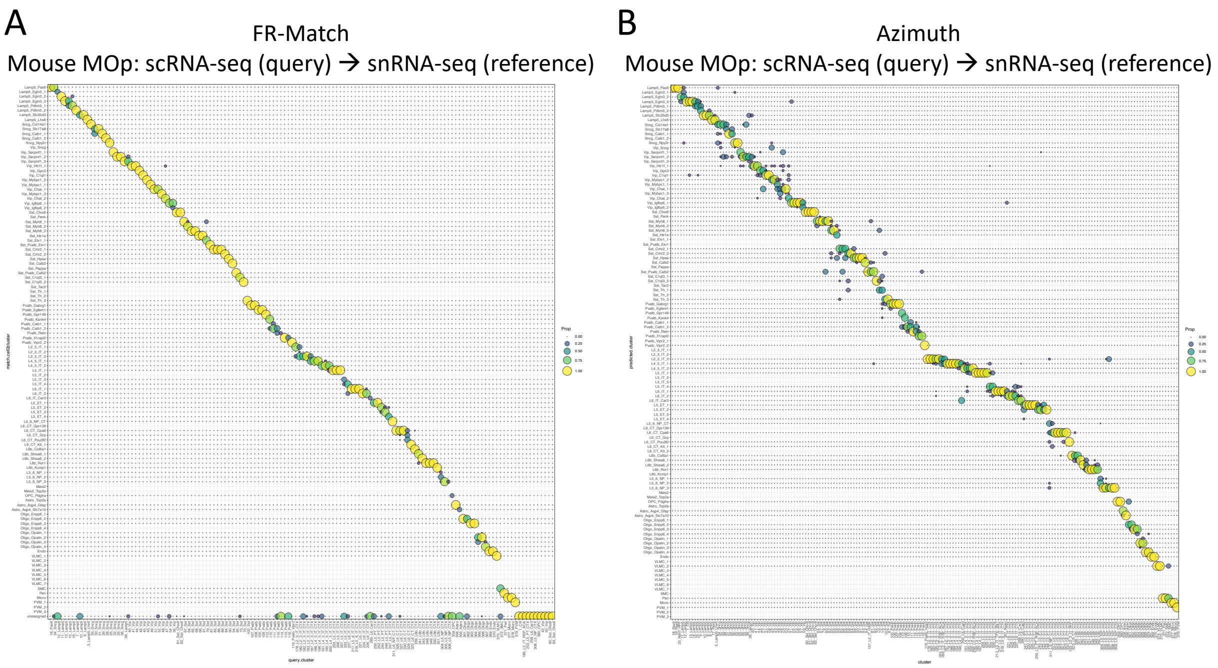

Supplementary Figure 3: FR-Match cell type matching performance in comparison with Azimuth. Matching results for matching cell types from scRNA-seq (query) to snRNA-seq (reference) datasets of mouse MOp cell types at the most granular cell type resolution using FR-Match (**A**) and Azimuth (**B**).

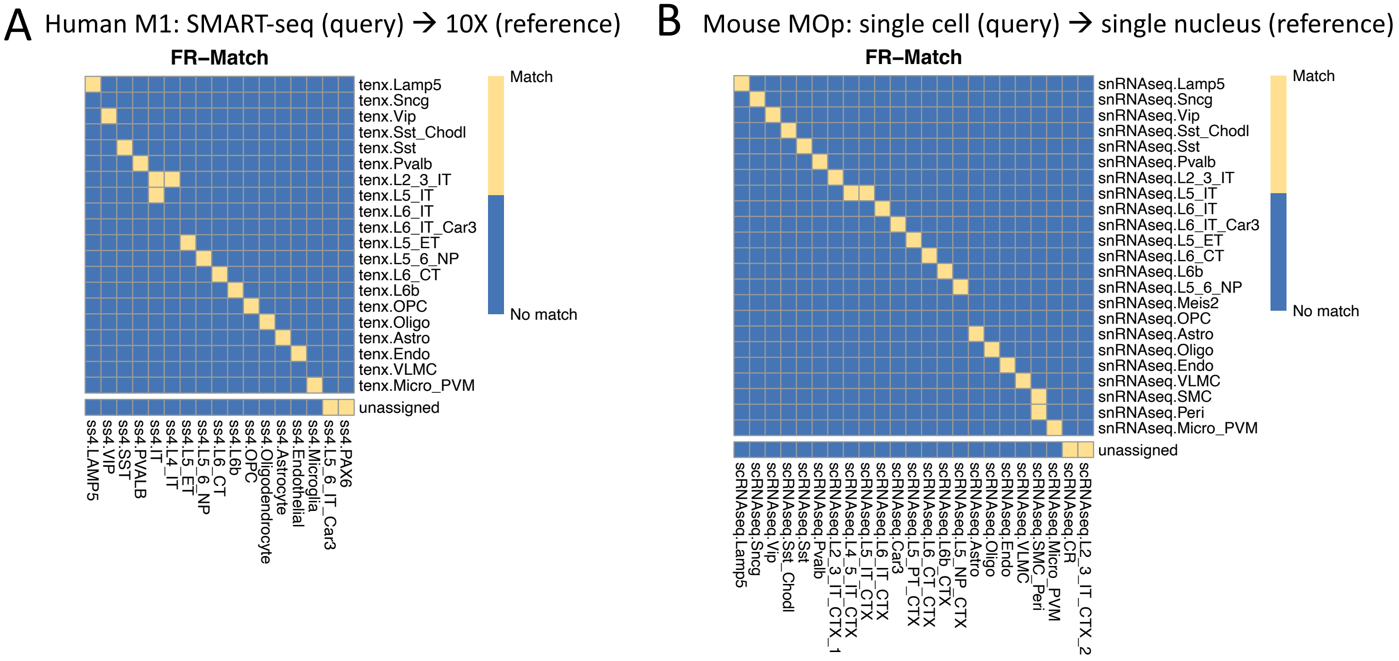

Supplementary Figure 4: FR-Match cluster-to-cluster matching results. A. Human M1 brain region *SMART-seq v4* (query) and *10X Chromium v3* (reference) datasets. **B.** Mouse MOp brain region single-*cell* RNA-seq (scRNA-seq) (query) and single-*nucleus* RNA-seq (snRNA-seq) (reference) 10X datasets. For both analyses, the same normalization steps were applied as in the corresponding cell-to-cluster FR-Match analyses; cosine distance was used. All one-to-one matches are the same as in the cell-to-cluster results. In panel (A), The one-to-many and many-to-one matches of the ss4.IT and ss4.L4_IT, and tenx.L2_3_IT and tenx.L5_IT types are the matching IT cells in the closest layers to each other. As mentioned in the main manuscript, the query ss4.IT is an agglomerated cluster where the cell-to-cluster approach detected matches to multiple reference IT clusters from upper layer to deep layer in decreasing proportions; and a small portion of the query ss4.IT cells were unassigned in the cell-level matching. The match ss4.L4_IT to tenx.L2_3_IT was also found by the cell-level matching, which is the match of upper layer IT cells in both query and reference datasets. The cluster-level unassigned ss4.L5_6_IT_Car3 and ss4.PAX6 query types were matched to tenx.L6_IT_Car3 and tenx.Sncg reference types in the cell-to-cluster results, which suggests that the cluster-level matching is more conservative as it considers the similarity of whole query clusters to whole reference clusters. For example, the ss4.PAX6 is a small cluster (28 cells in the query data) and tenx.Sncg is a populated cluster (895 cells in the reference data), and they are indeed similar inhibitory neurons. Therefore, at the subclass level, PAX6 cells are commonly clustered in the Sncg cell type in comprehensive reference datasets, which can be picked up by the cell-level matching but not by the cluster-level matching. In panel (B), the many-to-one match of scRNAseq.L4_5_IT_CTX and scRNAseq.L5_IT_CTX to snRNAseq.L5_IT was also found by the cell-to-cluster approach. The one-to-many match of scRNAseq.SMC_Peri to snRNAseq.SMC and snRNAseq.Peri is because the query cluster indeed contains both reference cell types, but the cluster-level approach was not able to split the composite query cluster into proportions of pure cell types. The cluster-level unassigned scRNAseq.CR query type was matched to snRNAseq.Lamp5 reference type in the cell-to-cluster results, also due to the high sensitivity of the cell-level approach for smaller sub-clusters (10 CR cells in the query data, which may be included in the major population of Lamp5 cell type with 2000 cells in the reference data). The cluster-level unassigned scRNAseq.L2_3_IT_CTX_2 query type was matched to multiple reference IT types mainly located in snRNAseq.L2_3_IT and snRNAseq.L5_IT, which may lead to inconclusive matching for the cluster-level approach.

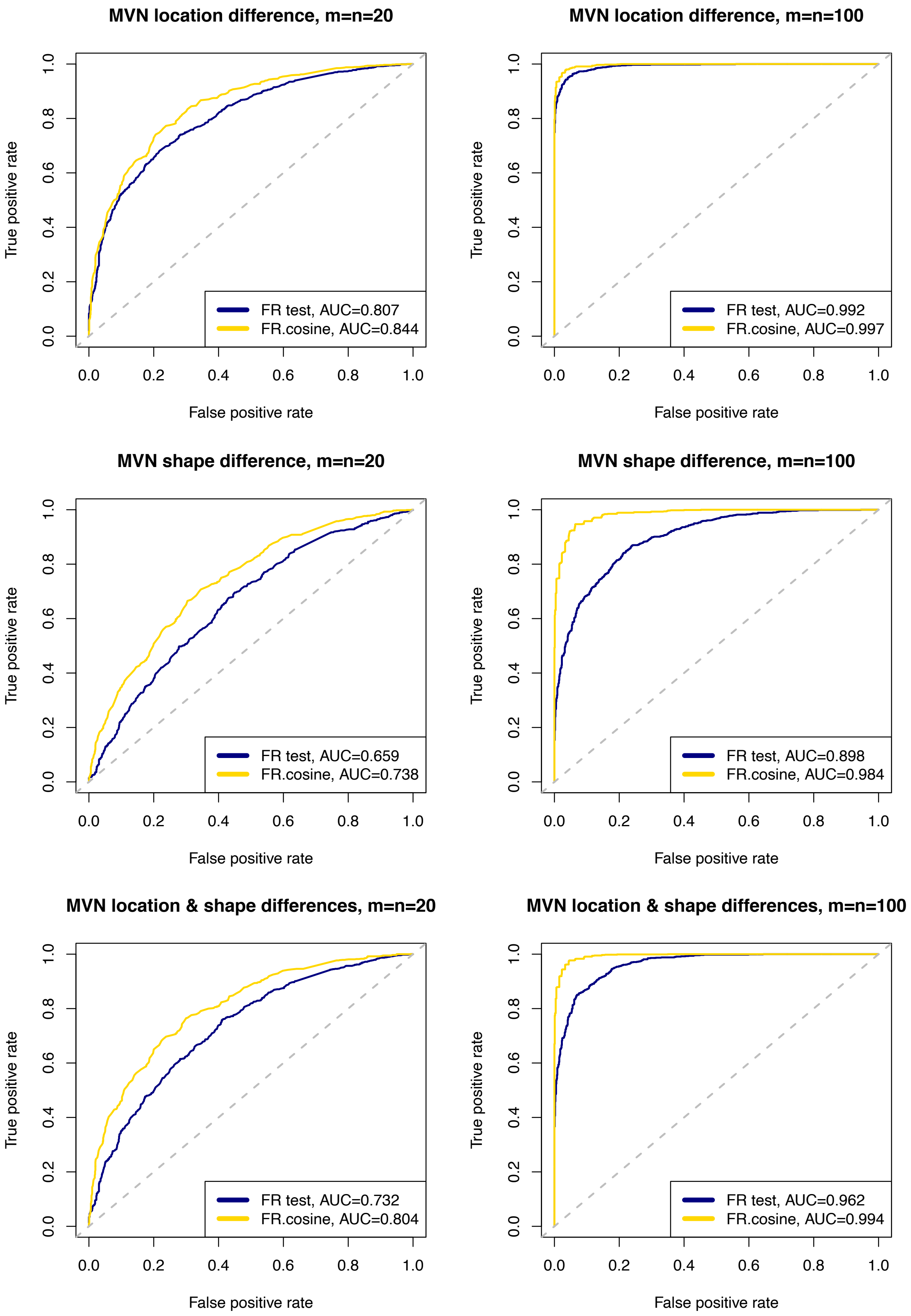

Supplementary Figure 5: Simulation performance of FR test using Euclidean (FR test) or cosine (FR.cosine) distance. The underlying distribution of the simulation study was a Multivariate Normal (MVN) distribution with location difference only (top), shape difference only (middle), and both location and shape differences (bottom). Small (left) and large (right) sample sizes are evaluated.
